# Association of self-reported musculoskeletal pain with school furniture suitability and daily activities among primary school and university students

**DOI:** 10.1371/journal.pone.0305578

**Published:** 2024-10-24

**Authors:** Nastja Podrekar Loredan, Dean Lipovac, Kaja Kastelic, Nejc Šarabon

**Affiliations:** 1 Faculty of Health Sciences, University of Primorska, Koper, Slovenia; 2 Hospital for the Treatment and Rehabilitation of Chronic Lung Diseases and Extended Hospital Treatment in Sežana, Sežana, Slovenia; 3 Institute of Criminology at the Faculty of Law, Ljubljana, Slovenia; 4 Faculty of Mathematics, Natural Sciences and Information Technologies, University of Primorska, Koper, Slovenia; 5 InnoRenew CoE, Izola, Slovenia; 6 Andrej Marusic Institute, University of Primorska, Koper, Slovenia; Bangladesh University of Engineering and Technology, BANGLADESH

## Abstract

Students spend a considerable amount of time in school. This study aimed to determine the prevalence of musculoskeletal pain and the association between the pain and suitability of school furniture and daily activities among primary school and university students. A total of 238 primary and university students participated in the study. The adapted Nordic questionnaire was used to assess pain prevalence, the BackPEI questionnaire was used to assess school-related factors, and student furniture mismatch calculations were performed to determine the anthropometric suitability of school furniture. Most students did not have a proper body posture while sitting, did not lift objects properly, and used TV and computer frequently. A high student-furniture mismatch was found for seat and desk height. The neck, lower back, shoulders, and upper back were the most affected body parts. Our study confirmed that musculoskeletal pain occurs in primary school students and increases with age, especially lower back pain. Proper backpack wearing was found to be an important factor in alleviating neck pain. Students who sat or lifted objects properly were more likely to report upper back pain, and students whose backrest height was appropriate were more likely to report lower back pain. Further efforts should be made to establish a comparable study protocol using objective methods to obtain more valid and reliable data to study school-related risk factors for musculoskeletal pain in students using prospective study protocols.

## Introduction

Musculoskeletal pain (MSP) is defined as acute or chronic pain that affects bones, muscles, ligaments, tendons, and even nerves, and is a common medical and socioeconomic problem worldwide [1]. Approximately 47% of the general population have experienced MSP, regardless of age, gender, or economic status [2]. Lower back pain (LBP) and neck pain have increased substantially over the past 25 years and are recognised as a leading cause of the number of years lived with disability in most countries around the world [3]. The majority of MSP data focuses on adults as the prevalence of MSP is thought to increase with age; however, the prevalence of LBP in adolescents is already similar to adults by the age of 18 [4]. In 2019, 34% of children in the United States reported suffering from LBP [5].

Studies identifying risk factors for MSP in children and adolescents are inconclusive [6–8], as the risk factors for MSP in school children are multifactorial and the cause of the pain often remains unknown [9]. For example, in the meta-analysis by Huguet et al. [9], high quality evidence suggested low socioeconomic status as the only risk factor for MSP. In another study, back pain was associated with psychological stress and female gender [10]. As children and adolescents spend a considerable amount of time at school each day, the associated risk factors should be considered.

Noll et al. [11] reported that back pain is associated with daily screen time, sitting posture when handwriting, computer use, and improper backpack carrying. Inappropriate school furniture has also been identified as a risk factor for MSP in children and adolescents [12]. It has been shown that neck pain is associated with a school desk that is too high, and shoulder pain is associated with a school desk that is too low [13–15]. On the other hand, Brewer et al. [16] found no association between the student-furniture mismatch and pain.

The studies conducted so far indicate an alarming prevalence of MSP in children and adolescents worldwide. However, only a limited number of studies are investigating the prevalence of pain in Slovenian children and adolescents. Moreover, the risk factors for the occurrence of pain in children and adolescents are not clear, and the studies conducted to date provide contradictory results. Studies that examined school-related risk factors such as school furniture, sitting posture, and duration of daily sitting do not provide conclusive results.

This cross-sectional study aimed to investigate the prevalence of MSP and the risk factors that may be associated with it in Slovenian primary school and university students. The study aimed to answer the following questions: i) What is the prevalence of MSP among Slovenian primary school and university students? ii) Are suitable school furniture and daily activities associated with MSP among Slovenian primary school and university students?

We hypothesized that students whose school furniture does not fit their body dimensions are more likely (odds ratio > 1.50, p < 0.05) to develop neck, shoulder, or lower or upper back pain. Further on, we hypothesized that students who do not sit, lift objects, or wear school bag properly are more likely (odds ratio > 1.50, p < 0.05) to develop neck, shoulder, or lower or upper back pain.

## Methods

Local schools were contacted to participate from 7^th^ to 23^rd^ December 2020. After school authorities accepted to collaborate in the study, a written invitation including all relevant information on the study has been sent to students and their parents on 12^th^ of January 2021. A signed informed consent has been obtained from students in February. For students aged less than 18, informed consent had to be signed also by their parents/legal guardians. The study was carried out between March and June 2021. The study was conducted in line with the latest revision of the Helsinki Declaration and approved by the Slovenian National Medical Ethics Committee (number of approval 0120-347/2018/3). The study protocol was pre-registered with clinical trials under the registration number ID NCT03653767. The data analysed in the study were completely anonymous.

### Participants

Students from one public primary school and one public faculty from the western part of Slovenia participated in this study. To be included in the study, participants 1) had to use school furniture (e.g., people who use a wheelchair did not fit inclusion criteria), 2) had to be involved in regular schooling process, and 3) must had been able to read and write in Slovenian language. All students who volunteered were generally healthy and had no physical or mental disabilities. A total of 272 primary (4th to 9th grade) and university (1st to 3rd year) students decided to participate in the study. The final sample consisted of 238 primary and university students because 17 students were not present on the day of the measurement and 17 students provided incomplete data. All students were briefed about the purpose of the study before the beginning of the measurements. In the article, the term “student” is used to refer to both primary school and university participants.

### Pain prevalence assessment

An adjusted Nordic Questionnaire [17] has been used to assess self-reported pain (S1 Questionnaire). Nordic Questionnaire has been psychometrically tested in several countries and populations as well as in the online context, displaying satisfactory properties, including good test-retest reliability, internal consistency, and construct validity [18–22]. The questionnaire consisted of a mannequin with labelled body parts (neck, shoulders, upper back, elbows, wrists, lower back, knees, and ankles). For each body part students answered questions regarding pain occurrence (in lifetime, in the last 12 months, in the last seven days, and on the day of the measurement), duration of pain, reduced everyday activities, and medication. Researchers were present when students answered the questionnaire and assisted if needed. Pain prevalence in the last 12 months in the area of neck, shoulders, upper back, and lower back was included in the further statistical analysis as these were the body parts with the highest pain prevalence and were most likely to be affected by the school related risk factors.

### Assessment of daily activities

For daily activities assessment, the Back Pain and Body Posture Evaluation Instrument (BackPEI) has been used. The questionnaire was developed to evaluate risk factors for back pain in school children and has shown sufficient reproducibility and validity [23]. A revised version of the questionnaire has been psychometrically examined on a sample of older adults, with the authors concluding that the tool is valid and reliable [24]. The questionnaire was translated into Slovene by K.K. and reviewed for its clarity by N.P.L. and N.S. The Slovenian version of the questionnaire follows the visual appearance and technical instructions of the original questionnaire. The BackPEI consists of six parts: (1) demographic data; (2) socio-economic status; (3) behavioral factors (physical activity, watching TV duration, using computer duration, etc.); (4) LBP in the last three months (occurrence, frequency, and intensity); (5) postural factors (sitting position while writing or using a computer or talking on the school furniture etc.); (6) genetic factors. Questions related to LBP were omitted from the questionnaire used in this research to avoid duplication of the questions with the adjusted Nordic Questionnaire.

### School furniture suitability measurements

Measurements of school furniture suitability consisted of school furniture measurements and measurements of students’ body dimensions. The measurements and calculations have been carried out according to Castellucci et al. [25]. In this study, suitability of seat and desk height, seat depth, and backrest height have been used as these furniture dimensions were the most unsuitable for students. Detailed results of the measurements are reported elsewhere [26].

### Statistical analysis

The data were processed and analyzed with R 4.0.2 (Team, 2021) using RStudio 1.4.1106 (RStudio Team, 2021) with the packages janitor [27], rstatix [28], DHARMa [29], gtsummary [30], and the collection of packages tidyverse [31]. Datasets and documentation of the analysis R are available online [32].

We analysed the associations between potential associated factors and pain prevalence with four instances of the binary logistic regression model. In all models, predictor variables (potential risk factors) included a selection of relevant participants’ characteristics, activities, and habits as well as the suitability of the school furniture (desk and seat characteristics) for participants’ body dimensions.

### Predictor and outcome variables

To simplify the data and make them more informative, some predictor variables were created by merging several variables, categorizing a continuous outcome, and/or reducing the number of categories within a variable.

Age was categorized in two groups (8–15 years and 18–35 years) to better represent the distinct age distributions of primary school and university participants. Sex was categorized as male or female.

Body mass index (BMI) was categorized according to standard classifications for adults (at least 18 years old) [33] and specific World Health Organization classifications for children [34]. Adults were classified as “underweight” (BMI < 18.5), “normal” (BMI > = 18.5 and < 25), “overweight” (BMI > = 25 and < 30), and “obese” (BMI > = 30). Children were classified as “underweight”, “normal”, “overweight”, or “obese” based on the different z-scores provided for each combination of age and sex (where more than -2 SD, +1 SD, and +2 SD away from the median BMI for specific age and sex was classified as “underweight”, “overweight”, and “obese”, respectively, with the rest of children classified as “normal”). The resulting classifications for both adults and children were then grouped into two categories (“overweight or obese” and “underweight or normal”), due to the low numbers of participants in the extreme BMI categories.

Based on the questionnaire used (BackPEI), we were unable to determine whether primary school participants achieved the weekly physical activity levels recommended by the World Health Organization for children and adolescents aged 5 to 17 years [35]. Therefore, we divided all participants (both primary school and university participants) into two groups based on the physical activity guidelines for adults, which recommend at least 150 minutes of moderate or vigorous physical activity per week [35]. Sport activities were thus classified as less than 150 min per week or 150 min per week or more. We hypothesized that primary school participants who did not achieve the adult recommendations would not achieve the child and adolescent recommendations.

The variable “sitting body posture” was created from three variables separately assessing the appropriateness of body posture when sitting in three different situations: at a school desk, during a conversation, and while using a computer. This variable had two outcomes: i) “proper never” and ii) “proper at least sometimes”. The participants were categorized as “proper never” if they marked improper body posture for all sitting conditions. If this was not the case, they were categorized as “proper at least sometimes”.

The variables “lifting objects” and “bag carrying” were given values “improper” or “proper”, based on participants’ selection of one of the several images that best represents their way of lifting objects and bag carrying, respectively. The variable “bag type” was given values “backpack” or “other bag type”, based on the participants’ selection of one of five possible bag types (in addition to the option “other”).

The variable “TV and computer use” was created from two variables separately assessing the frequency of TV and computer use in number of hours per day. If participants used the computer and TV for 1 hour or less (each), they were classified as “2h or less” in the variable “TV and computer use per day”. Conversely, if they reported 2 or more hours in either computer or TV use, they were classified in the variable as “More than 2h”.

The four variables on suitability of 1) seat height, 2) seat depth, 3) desk height, and 4) backrest height each had two possible outcomes: suitable or not suitable, based on the calculations specified by Castellucci et al. [25].

Outcome variables (pain prevalence) in the regression models included the prevalence of the pain (present or not present) in the last 12 months at four different body parts (neck, shoulder, upper back, lower back), with the pain in each body part serving as an outcome in one of the four regression models. The results of the regression analyses are reported as odds ratios with 95% confidence intervals.

### Sample size, number of regression predictors, and model diagnostics

We limited the total number of predictors included in the regression analyses to 12 (binary) predictor variables, based on the calculation method by Smeden et al. [36] and the accompanying software [36]. Considering the number of predictors, fraction of events in the outcome, and sample size, the calculation suggests that the estimated prediction error of our least precise regression model is acceptable (specifically, the square root of the mean squared prediction error (rMPSE) is approximately 0.1). The variables on parents’ back pain and parents’ education were not included as predictor variables because we wanted to adhere to the above-mentioned limit of 12 included predictors and because around 20% of participants did not know the answers to the questions on parents’ back pain and education.

Model diagnostics were carried out using the R package DHARMa [29], which analyses residuals of various types of statistical models using a simulation-based method. None of the presented models had issues with fit to the data.

## Results

### Sample characteristics

The mean (± standard deviation) age of primary school students, university students, and the total sample was 11.0 ± 1.6, 20.2 ± 1.9, and 16.5 ± 4.9 years, respectively. The mean (± standard deviation) body height and body weight of primary school students, university students, and the total sample were 153 ± 11 cm and 44.6 ± 11.7 kg, 170 ± 9 cm and 66.0 ± 11.6 kg, and 163 ± 13 cm and 57.4 ± 15.7 kg, respectively.

The remaining characteristics of participants are presented in Table 1, first separately for each school and then together for the entire sample of participants. About two-thirds of the entire sample of participants were female and about three-quarters were underweight or had normal weight (2 university students and 52 primary school students were underweight). Most of the participants did not meet weekly recommendations for sports activity, did not have a proper body posture while sitting, did not lift objects properly, and used TV and computer frequently. The most frequently used bag type among the participants was the backpack, and most participants carried their bag properly. Seat height and desk height were suitable for the minority of the entire sample, while seat depth and backrest height were suitable for most of the participants.

**Table 1:** Participant characteristics separately for primary school and university and combined for the entire sample.

Characteristic	Primary school (4^th^ to 9^th^ grade)	University (1^st^ to 3^rd^ year)	Total
Age group			
8 to 15 years	96 (100%)	0 (0%)	96 (40%)
18 to 35 years	0 (0%)	142 (100%)	142 (60%)
Sex			
Female	54 (56%)	102 (72%)	156 (66%)
Male	42 (44%)	40 (28%)	82 (34%)
Body mass index (BMI)			
Overweight or obese	31 (32%)	26 (18%)	57 (24%)
Underweight or normal	65 (68%)	116 (82%)	181 (76%)
Sport duration per week			
Less than 150 min/week	88 (92%)	61 (43%)	149 (63%)
150 min/week or more	8 (8.3%)	81 (57%)	89 (37%)
Sitting body posture			
Proper never	72 (75%)	112 (79%)	184 (77%)
Proper at least sometimes	24 (25%)	30 (21%)	54 (23%)
Lifting objects			
Improper	84 (88%)	71 (50%)	155 (65%)
Proper	12 (12%)	71 (50%)	83 (35%)
Bag type			
Backpack	90 (94%)	102 (72%)	192 (81%)
Other bag type	6 (6%)	40 (28%)	46 (19%)
Bag carrying			
Improper	23 (24%)	49 (35%)	72 (30%)
Proper	73 (76%)	93 (65%)	166 (70%)
TV and computer use per day			
More than 2h	60 (62%)	92 (65%)	152 (64%)
2h or less	36 (38%)	50 (35%)	86 (36%)
Furniture suitability			
Seat height (% suitable)	13 (14%)	66 (46%)	79 (33%)
Seat depth (% suitable)	71 (74%)	103 (73%)	174 (73%)
Desk height (% suitable)	16 (17%)	74 (52%)	90 (38%)
Backrest height (% suitable)	49 (51%)	136 (96%)	185 (78%)

### Pain prevalence

**Fig 1:**
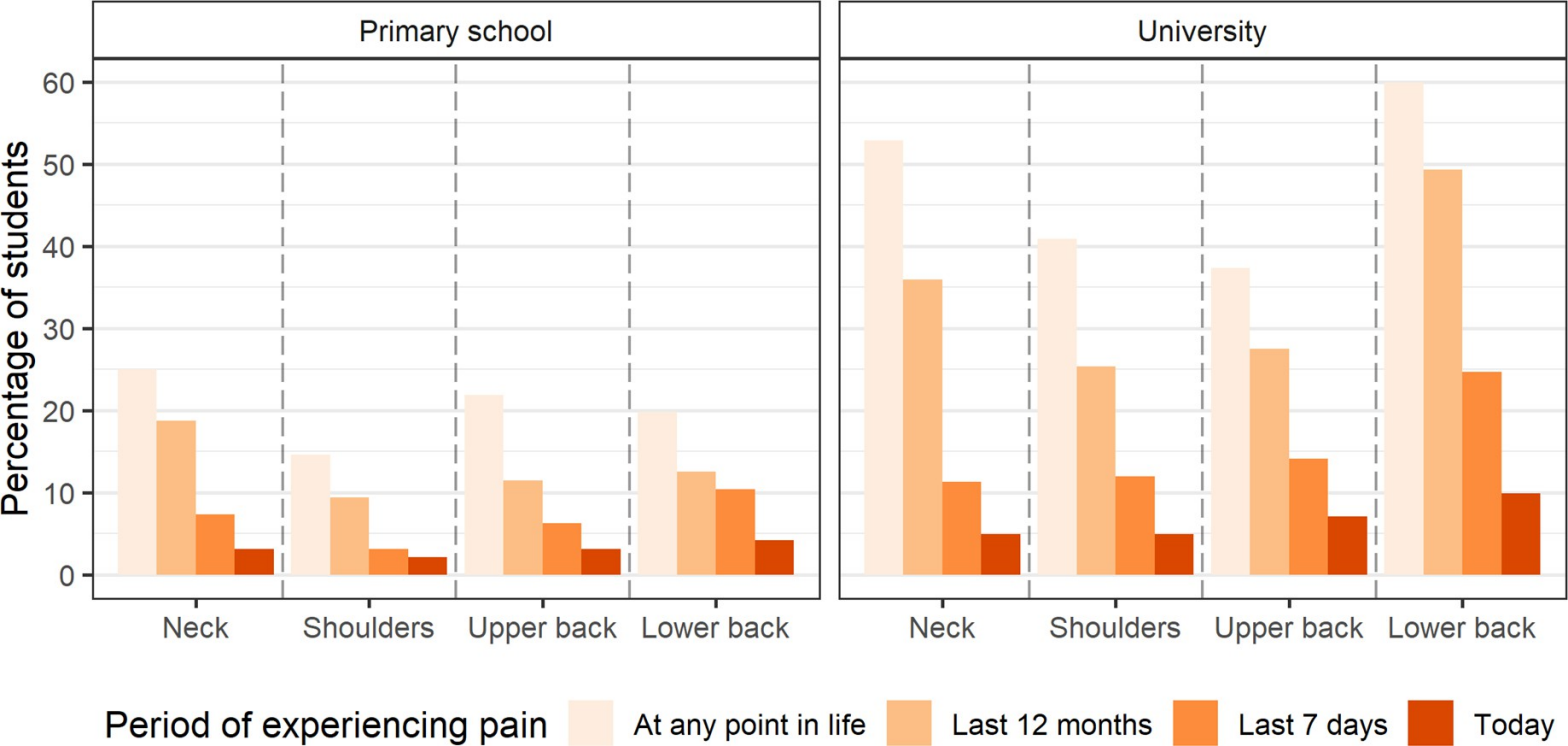
Pain prevalence in different body parts at different time periods.

Pain prevalence (separated by school) in different body parts at different time periods is presented in Fig 1. Between 9% and 19% of primary school students and 25% and 49% of university students experienced pain at some point in their life in the four body parts. The pain prevalence in both groups of students steadily decreased with every narrower time period, with the highest pain prevalence reported for “any point in life”, followed by “last 12 months”, “last 7 days”, and “today”. In the primary school students, the pain prevalence was somewhat higher in the neck compared to the other body parts, while the university students reported a somewhat higher pain prevalence in the neck and lower back compared to the shoulders and upper back. Detailed results regarding the pain prevalence in different body parts at different time periods and in different body parts at different time periods across sexes for the entire set of participants and separated by school are presented in S1 and S2 Tables.

When asked about the longest continuous pain experienced in each of the four body parts, between 0% and 1% of primary school students and between 1% and 3% of university students reported that their pain had at one point lasted more than 30 days continuously. When asked about the pain-related disturbance connected to each of the four body parts, between 2% and 12% of primary school students and between 4% and 16% of university students reported that the pain had obstructed their activities in the last 12 months; between 2% and 6% of primary school students and between 2% and 7% of university students reported they had visited a doctor in the last 12 months because of the pain; and between 1% and 3% of primary school students and between 1% and 6% of university students reported they had taken medication in the last 12 months because of the pain.

### Associations between participant characteristics and pain in the last 12 months

S3 Table presents the percentages of people reporting pain in the last 12 months for each of the analysed body parts, separately for all predictor variables that were included in the regression analyses.

Table 2 displays the results of the four regression analyses with the outcomes of pain prevalence in the last 12 months in the 1) neck, 2) shoulders, 3) upper back, and 4) lower back. Out of the 12 total predictors, seven were not statistically significant predictors in any of the regression models. Each of the remaining five variables was a statistically significant predictor in only one of the four regression models. None of the included predictor variables were significantly associated with the pain prevalence in the shoulders, while significant associations were found between one or two predictors and pain in each of the three remaining body parts.

**Table 2:** Results of the logistic regression models with pain (in the last 12 months) in different body parts as dependent variables.

	Neck		Shoulders		Upper back		Lower back	
	Odds ratio [95% confidence interval]	p value	Odds ratio [95% confidence interval]	p value	Odds ratio [95% confidence interval]	p value	Odds ratio [95% confidence interval]	p value
**(Intercept)**	0.28 [0.09, 0.85]	0.029	0.11 [0.03, 0.42]	0.002	0.09 [0.02, 0.35]	0.001	0.06 [0.01, 0.23]	< 0.001
**Age: 18 or more**	1.97 [0.82, 4.89]	0.133	3.04 [1.01, 10.13]	0.057	2.13 [0.80, 5.94]	0.137	**5.66** **[2.31, 14.86]**	**< 0.001**
**Sex: Male**	0.99 [0.48, 2.04]	0.975	1.39 [0.60, 3.25]	0.441	0.94 [0.41, 2.14]	0.886	1.18 [0.54, 2.56]	0.676
**BMI: Underweight or normal**	1.05 [0.51, 2.25]	0.896	0.87 [0.39, 2.06]	0.749	0.76 [0.34, 1.76]	0.509	0.47 [0.21, 1.00]	0.052
**Sports: As recommended or more**	1.17 [0.57, 2.39]	0.660	1.85 [0.84, 4.14]	0.132	0.57 [0.25, 1.26]	0.172	1.01 [0.5, 2.03]	0.986
**Sitting body posture: Proper at least sometimes**	1.01 [0.48, 2.06]	0.987	0.74 [0.30, 1.69]	0.499	**2.34** **[1.09, 4.97]**	**0.027**	0.98 [0.46, 2.05]	0.952
**Lifting objects: Proper**	1.08 [0.55, 2.11]	0.818	1.14 [0.53, 2.42]	0.729	**2.17** **[1.04, 4.58]**	**0.040**	0.85 [0.43, 1.64]	0.625
**Bag carrying: Proper**	**0.43** **[0.23, 0.82]**	**0.010**	0.89 [0.43, 1.92]	0.768	0.72 [0.35, 1.50]	0.381	0.74 [0.39, 1.43]	0.371
**TV and computer use: Little**	0.76 [0.40, 1.43]	0.401	1.18 [0.58, 2.39]	0.641	0.84 [0.40, 1.70]	0.630	1.03 [0.54, 1.98]	0.919
**Seat height: Suitable**	1.59 [0.78, 3.26]	0.205	0.83 [0.36, 1.87]	0.658	1.21 [0.54, 2.68]	0.638	0.94 [0.46, 1.91]	0.856
**Seat depth: Suitable**	1.65 [0.80, 3.53]	0.184	1.03 [0.47, 2.36]	0.940	1.20 [0.55, 2.75]	0.660	1.88 [0.90, 4.06]	0.101
**Desk height: Suitable**	0.61 [0.31, 1.2]	0.154	1.08 [0.51, 2.33]	0.836	1.40 [0.67, 2.94]	0.371	0.69 [0.36, 1.31]	0.259
**Backrest height: Suitable**	1.15 [0.44, 3.03]	0.776	0.69 [0.21, 2.33]	0.546	1.59 [0.50, 5.67]	0.447	**5.71** **[1.82, 22.00]**	**0.005**

Significant predictors (p < 0.05) are shown in bold.

Age was a strong predictor for LBP as university students had significantly greater odds of having LBP compared to primary school students. Participants who reported carrying their bag properly were less likely to report pain in the neck in the last 12 months, compared with the reference group. Those who reported sitting properly at least sometimes or lifting objects properly had greater odds of reporting pain in the upper back in the last 12 months (compared with the reference group). Finally, participants who had a backrest height suitable for their body dimensions were much more likely to report pain in the lower back, compared with the reference group.

## Discussion

This study aimed to determine the prevalence of MSP and to investigate the association between MSP, school furniture suitability, and daily activities among Slovenian primary school and university students. The highest prevalence of pain in primary school students was observed in the neck area, while the university students most frequently reported LBP. One hypothesis was confirmed: Students who carried their school bags properly, using both straps, were less likely to suffer from neck pain. Other hypotheses were rejected, as we could not find an association between unsuitable furniture and MSP, nor between improper sitting posture or improper lifting technique and MSP.

### Pain prevalence

Because of the negative impact of MSP on individuals’ quality of life, health burden, and economic costs, understanding and managing pain in children and adolescents is an important starting point to reduce the development of MSP in later adulthood. Most studies examining the occurrence of pain in children and adolescents focus on the prevalence of LBP [4], while studies examining pain in other body regions are rare. In our study, primary school students most frequently reported suffering from neck pain (18.8%), followed by LBP, upper back pain, and shoulder pain in the last 12 months. Comparing our results with the existing literature, we can conclude that the prevalence of MSP is slightly lower in Slovenian primary school students compared to primary school students worldwide. Santos et al. [37] found a monthly prevalence of LBP of 27.3% in Brazilian primary school students aged 6 to 12 years, similarly the monthly prevalence of LBP in Iranian students (7 to 12 years) was 26.6% [38]. Similar results to ours were found in students (11 to 14 years) from the United Kingdom with a monthly pain prevalence of 27% for neck pain, 18% for upper back pain, and 22% for LBP [39]. Half of Slovenian university students (49.3%) reported suffering from LBP in the last 12 months and 25.1% in the last seven days. This is similar to reports of Norway students, where half of them experiences chronic pain in at least one part of the body [40], while higher prevalence of pain is reported by Turkish (73.3% in the last month) and Nigerian (67.3% in the last seven days) students aged 15 to 25 years [13, 41]. Although we found a lower pain prevalence in Slovenian primary school and university students compared to students from other countries, differences in time-prevalence and methodologies should be taken into consideration.

The literature shows that MSP is already present in six-year-old children [42] and increases with age [43, 44]. Indeed, in our study, university students reported a significantly higher prevalence of MSP, especially LBP, compared with primary school students. Brazilian adolescents were also found to have a significant increase in back pain over three years [43]. In our sample of university students, female participants reported LBP and neck pain more frequently than male subjects. A similar outcome was observed among Norway university students, where female students reported significantly more pain compared to male students [40].

### Association between pain and school furniture suitability

Studies suggest that inadequate school furniture may increase student discomfort and pain [14, 15, 39]. Inappropriate school desk was associated with musculoskeletal pain, specifically, neck pain was associated with a desk that is either too high [15] or too low [14]. A too low school desk was also associated with LBP [38] and shoulder pain [15]. Moreover, Rezapur-Shahkolai et al. [38] and Ayed et al. [14] reported too low backrests to be associated with LBP. In contrast, we found that the adequate backrest height was associated with greater odds for LBP. This could be explained by the fact that backrest height was suitable for almost all university students (96%) who simultaneously also had a high LBP prevalence in the last 12 months (49.3%), which could lead to a misleading result. Otherwise, in our study, no significant association between an inappropriate school desk or chair and musculoskeletal pain was found, which is consistent with the findings of Skoffer [45] and Brewer et al. [16].

Studies investigating the impact of school furniture on MSP in students differ in study protocols. In some studies, the suitability of school furniture was assessed subjectively using questionnaires [14, 38, 39]. On the other hand, we calculated the school-furniture mismatch based on students’ anthropometric measurements and furniture dimensions using the equations of Castellucci et al. [25]. Pain prevalence is also reported differently in the studies; Gheysvandi et al. [15] referred to pain prevalence in the last month, Ayed et al. [14] reported pain prevalence in the last three months, while our study used pain prevalence in the last 12 months.

### Association between pain and daily activities

Participants who reported sitting or lifting objects properly at least sometimes (participants marked improper body posture for two or less sitting conditions) had about two times greater odds of reporting upper back pain in the past 12 months. This finding is conflicting with the existing literature, as Ozdemir et al. [41] reported that 38.1% of Turkish students attributed their MSP to their poor sitting posture at school. Furthermore, in a study by Minghelli et al. [46], students who adopted poor sitting posture had 2.5-times (95% CI 1.91–3.2; p < 0.001) higher probability of developing LBP. Sitting posture and posture during computer use were also found to be risk factors for back pain in Brazilian students [11].

We could explain our results by the fact that students who do not have pain do not pay attention to their sitting posture or lifting techniques. On the other hand, students who already have back pain start paying attention to their posture while sitting or try to lift objects in a more correct way to avoid aggravating the pain. Similar results were reported by Saraceni et al. [47], who found that workers with LBP perform lifting with less lumbar flexion than workers without LBP, although it is generally recommended to avoid severe lumbar flexion. Somewhat comparable results were reported by Richards et al. [48], who also found that sitting neck posture was not a risk factor for neck pain in men, while in women a more relaxed posture, such as a slumped thorax and head bent forward, was actually protective against neck pain compared to an upright posture.

As the findings on posture and associated MSP are inconclusive, further research with more detailed questions on the occurrence of pain and postural behavior is needed to investigate the impact of sitting posture on the occurrence of pain in students. In addition, research on the occurrence of pain and associated posture should be rethought to define what is the cause and what is the consequence, using prospective cohort studies.

To mitigate the risk of MSP, adequate physical fitness is also an important factor [49]. In our study, a total of 63% of students were physically active for less than 150 minutes per week, with primary students being less physically active compared with university students. We did not find that physical activity level, TV, and computer use significantly influenced the occurrence of pain. However, this was not the case in other studies. Obese and overweight children might be less physically active [50] and, as reported by Elgaeva et al. [51], children with a higher BMI have a higher risk for back pain. Further on, spending more than 3 hours per day watching TV increased the chance of LBP among Brazilian children (OR = 7.97, CI 95% = 1.957–32.515, p = 0.004 [37, 43]. These differences could be due to the differences in study design, lifestyle, and cultural differences among participants. However, our results are to some extent comparable to those of Ozdemir et al. [41], who also found no association between computer use and the occurrence of pain.

### Strengths and limitations

The present study has several strengths. Measurement protocol was standardized across all study participants. Student-furniture mismatch was calculated based on the anthropometric and school furniture measurements using standardised tools. In our regression analyses, we controlled for several confounding variables that could influence the outcomes, and our regression models showed good fit to the data.

The study also has limitations. The sample size was not nationally representative because all participants were from the western region of Slovenia. University students were all Kinesiology or Physiotherapy students, which could influence their levels of physical activity and the prevalence of MSP, which limits the generalizability of our findings. Physical activity, sitting posture, and lifting techniques were self-reported, and the questionnaire asked only about sport-related physical activity, potentially leading to less accurate data.

### Conclusion

Risk factors for MSP in students are complex and often remain unknown. Prevention and management of MSP is an important aspect of student health and well-being as it not only impacts students’ physical, emotional, and developmental well-being, but also affects their social networks, as children with chronic pain have fewer friends and are more likely to be victims of peer victimization [52, 53].

Given the paucity of data on pain prevalence among Slovenian primary school and university students, the results of this study are informative for clinicians. Our results suggest that pain prevalence is somewhat higher among females, indicating a potential need for gender-tailored interventions. The main findings of the study are the following:

- Neck pain (18.8%), LBP, upper back pain, and shoulder pain were most frequently reported by primary school students, in the decreasing order of prevalence.
- LBP (49.3%), neck pain, upper back pain, and shoulder pain were most frequently reported by university students, in the decreasing order of prevalence.
- University students had five times greater odds to suffer from LBP than primary school students.
- Females reported LBP, upper back and neck pain more frequently compared to males when looking at all participants together.
- Students who wear their backpacks properly, using both straps, were less likely to suffer from neck pain.
- Students who sit and lift objects properly were more likely to experience upper back pain.

As we found conflicting results regarding the relationship between pain and proper sitting posture and lifting techniques, our results do not have immediate clinical implications. Further studies are needed to better understand the role of school furniture in students’ posture and musculoskeletal pain.

## Supporting information

S1 Table Pain prevalence in different body parts at different time periods for the entire set of participants and separated by school. (DOCX)

S2 Table Pain prevalence in different body parts at different time periods across sexes for the entire set of participants and separated by school. (DOCX)

S3 Table Pain prevalence (in the last 12 months) in different body parts across different regression predictors and their values. (DOCX)

S1 Questionnaire Adjusted Nordic questionnaire. (PDF)
