# Supplementary material for: Association of self-reported musculoskeletal pain with school furniture suitability and daily activities among primary school and university students
Source: PLoS One. 2024 Oct 24;19(10):e0305578. doi: 10.1371/journal.pone.0305578 (PMC11500950; doi:10.1371/journal.pone.0305578)
Supplement: S1 Table — (DOCX) [file pone.0305578.s001.docx]

S1 table: Pain prevalence in different body parts at different time periods for the entire set of participants and separated by school.

| Body part | School | Pain prevalence [%] | | | |
| --- | --- | --- | --- | --- | --- |
|  |  | At any point in life | Last 12 months | Last 7 days | Today |
| Neck | Both (all subjects) | 41.60 | 28.99 | 9.66 | 4.20 |
| Shoulders | Both (all subjects) | 30.25 | 18.91 | 8.40 | 3.78 |
| Upper back | Both (all subjects) | 31.09 | 21.01 | 10.92 | 5.46 |
| Lower back | Both (all subjects) | 43.70 | 34.45 | 18.91 | 7.56 |
| Neck | Primary school | 25.00 | 18.75 | 7.29 | 3.12 |
| Shoulders | Primary school | 14.58 | 9.38 | 3.12 | 2.08 |
| Upper back | Primary school | 21.88 | 11.46 | 6.25 | 3.12 |
| Lower back | Primary school | 19.79 | 12.50 | 10.42 | 4.17 |
| Neck | University | 52.82 | 35.92 | 11.27 | 4.93 |
| Shoulders | University | 40.85 | 25.35 | 11.97 | 4.93 |
| Upper back | University | 37.32 | 27.46 | 14.08 | 7.04 |
| Lower back | University | 59.86 | 49.30 | 24.65 | 9.86 |
