# Supplementary material for: Association of self-reported musculoskeletal pain with school furniture suitability and daily activities among primary school and university students
Source: PLoS One. 2024 Oct 24;19(10):e0305578. doi: 10.1371/journal.pone.0305578 (PMC11500950; doi:10.1371/journal.pone.0305578)
Supplement: S2 Table — (DOCX) [file pone.0305578.s002.docx]

S2 table: Pain prevalence in different body parts at different time periods across sexes for the entire set of participants and separated by school.

| Body part | School | Sex | Pain prevalence [%] | | | |
| --- | --- | --- | --- | --- | --- | --- |
|  |  |  | At any point in life | Last 12 months | Last 7 days | Today |
| Neck | Both (all subjects) | Female | 43.59 | 30.77 | 9.62 | 3.85 |
| Neck | Both (all subjects) | Male | 37.80 | 25.61 | 9.76 | 4.88 |
| Shoulders | Both (all subjects) | Female | 30.77 | 17.95 | 8.33 | 4.49 |
| Shoulders | Both (all subjects) | Male | 29.27 | 20.73 | 8.54 | 2.44 |
| Upper back | Both (all subjects) | Female | 32.05 | 21.79 | 10.90 | 5.13 |
| Upper back | Both (all subjects) | Male | 29.27 | 19.51 | 10.98 | 6.10 |
| Lower back | Both (all subjects) | Female | 47.44 | 37.18 | 20.51 | 7.05 |
| Lower back | Both (all subjects) | Male | 36.59 | 29.27 | 15.85 | 8.54 |
| Neck | Primary school | Female | 24.07 | 16.67 | 7.41 | 1.85 |
| Neck | Primary school | Male | 26.19 | 21.43 | 7.14 | 4.76 |
| Shoulders | Primary school | Female | 14.81 | 9.26 | 1.85 | 1.85 |
| Shoulders | Primary school | Male | 14.29 | 9.52 | 4.76 | 2.38 |
| Upper back | Primary school | Female | 22.22 | 11.11 | 1.85 | 0.00 |
| Upper back | Primary school | Male | 21.43 | 11.90 | 11.90 | 7.14 |
| Lower back | Primary school | Female | 20.37 | 12.96 | 11.11 | 1.85 |
| Lower back | Primary school | Male | 19.05 | 11.90 | 9.52 | 7.14 |
| Neck | University | Female | 53.92 | 38.24 | 10.78 | 4.90 |
| Neck | University | Male | 50.00 | 30.00 | 12.50 | 5.00 |
| Shoulders | University | Female | 39.22 | 22.55 | 11.76 | 5.88 |
| Shoulders | University | Male | 45.00 | 32.50 | 12.50 | 2.50 |
| Upper back | University | Female | 37.25 | 27.45 | 15.69 | 7.84 |
| Upper back | University | Male | 37.50 | 27.50 | 10.00 | 5.00 |
| Lower back | University | Female | 61.76 | 50.00 | 25.49 | 9.80 |
| Lower back | University | Male | 55.00 | 47.50 | 22.50 | 10.00 |
