# Supplementary material for: Association of self-reported musculoskeletal pain with school furniture suitability and daily activities among primary school and university students
Source: PLoS One. 2024 Oct 24;19(10):e0305578. doi: 10.1371/journal.pone.0305578 (PMC11500950; doi:10.1371/journal.pone.0305578)
Supplement: S3 Table — (DOCX) [file pone.0305578.s003.docx]

S5 table: Pain prevalence (in the last 12 months) in different body parts across different regression predictors and their values.

| **Predictor** | **Predictor value** | **Pain prevalence in the last 12 months [%]** | | | |
| --- | --- | --- | --- | --- | --- |
|  |  | **Lower back** | **Neck** | **Shoulders** | **Upper back** |
| Age group | 17 or less | 12.50 | 18.75 | 9.38 | 11.46 |
| Age group | 18 or more | 49.30 | 35.92 | 25.35 | 27.46 |
| Backrest suitability | No | 7.55 | 20.75 | 13.21 | 9.43 |
| Backrest suitability | Yes | 42.16 | 31.35 | 20.54 | 24.32 |
| Bag carrying | Improper | 41.67 | 41.67 | 20.83 | 25.00 |
| Bag carrying | Proper | 31.33 | 23.49 | 18.07 | 19.28 |
| Body mass index (BMI) | Overweight or obese | 36.84 | 26.32 | 19.30 | 21.05 |
| Body mass index (BMI) | Underweight or normal | 33.70 | 29.83 | 18.78 | 20.99 |
| Desk height suitability | No | 30.41 | 29.73 | 16.22 | 17.57 |
| Desk height suitability | Yes | 41.11 | 27.78 | 23.33 | 26.67 |
| Lifting objects | Improper | 29.68 | 26.45 | 15.48 | 15.48 |
| Lifting objects | Proper | 43.37 | 33.73 | 25.30 | 31.33 |
| School | Primary school | 12.50 | 18.75 | 9.38 | 11.46 |
| School | University | 49.30 | 35.92 | 25.35 | 27.46 |
| Seat depth suitability | No | 28.12 | 23.44 | 20.31 | 20.31 |
| Seat depth suitability | Yes | 36.78 | 31.03 | 18.39 | 21.26 |
| Seat height suitability | No | 30.82 | 25.16 | 16.98 | 18.87 |
| Seat height suitability | Yes | 41.77 | 36.71 | 22.78 | 25.32 |
| Sex | Female | 37.18 | 30.77 | 17.95 | 21.79 |
| Sex | Male | 29.27 | 25.61 | 20.73 | 19.51 |
| Sitting body posture | Proper never | 35.33 | 29.35 | 19.57 | 18.48 |
| Sitting body posture | Proper at least sometimes | 31.48 | 27.78 | 16.67 | 29.63 |
| Sport duration per week | Less than recommended | 28.19 | 25.50 | 12.75 | 19.46 |
| Sport duration per week | As recommended or more | 44.94 | 34.83 | 29.21 | 23.60 |
| TV and computer use per day | A lot | 34.87 | 30.26 | 17.76 | 21.71 |
| TV and computer use per day | Little | 33.72 | 26.74 | 20.93 | 19.77 |
