# Supplementary material for: Association of self-reported musculoskeletal pain with school furniture suitability and daily activities among primary school and university students
Source: PLoS One. 2024 Oct 24;19(10):e0305578. doi: 10.1371/journal.pone.0305578 (PMC11500950; doi:10.1371/journal.pone.0305578)
Supplement: S1 Questionnaire — (PDF) [file pone.0305578.s004.pdf]

S3 questionnaire: Adjusted Nordic questionnaire (English and Slovenian versions)

		Have you ever had problems (such as pain, discomfort, numbness) in the area:	How old were you when the problem first appeared?	Have you had any problems in the area in the past 12 months:	In the last 12 months, have any of these problems prevented you from doing everyday activities (e.g. hobbies, work, tidying up)?	Have you seen a doctor in the past 12 months for a problem in the area?	You have been taking medication for problems in the area:	Have you had any problems in the area in the past 7 days:	Are you having problems in the area today:	What was the maximum duration of continuous pain?
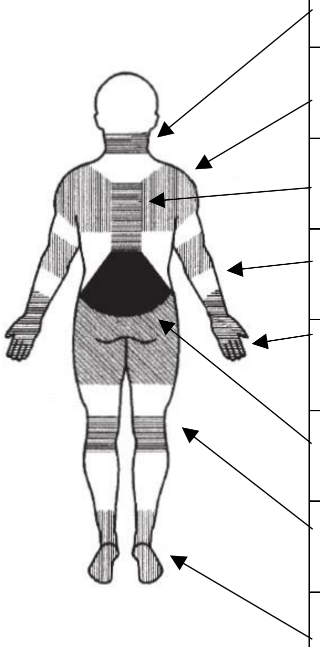	**NECK**	☐ yes ☐ no	__years	☐ yes ☐ no	☐ yes ☐ no	☐ yes ☐ no	☐ yes ☐ no	☐ yes ☐ no	☐ yes ☐ no	☐ 0 days, ☐ 1-7 days, ☐ 8-30 days ☐ > 30 days
	**SHOULDERS**	☐ yes ☐ no	__years	☐ yes ☐ no	☐ yes ☐ no	☐ yes ☐ no	☐ yes ☐ no	☐ yes ☐ no	☐ yes ☐ no	☐ 0 days, ☐ 1-7 days, ☐ 8-30 days ☐ > 30 days
	**UPPER BACK**	☐ yes ☐ no	__years	☐ yes ☐ no	☐ yes ☐ no	☐ yes ☐ no	☐ yes ☐ no	☐ yes ☐ no	☐ yes ☐ no	☐ 0 days, ☐ 1-7 days, ☐ 8-30 days ☐ > 30 days
	**ELBOWS**	☐ yes ☐ no	__years	☐ yes ☐ no	☐ yes ☐ no	☐ yes ☐ no	☐ yes ☐ no	☐ yes ☐ no	☐ yes ☐ no	☐ 0 days, ☐ 1-7 days, ☐ 8-30 days ☐ > 30 days
	**WRISTS**	☐ yes ☐ no	__years	☐ yes ☐ no	☐ yes ☐ no	☐ yes ☐ no	☐ yes ☐ no	☐ yes ☐ no	☐ yes ☐ no	☐ 0 days, ☐ 1-7 days, ☐ 8-30 days ☐ > 30 days
	**LOWER BACK**	☐ yes ☐ no	__years	☐ yes ☐ no	☐ yes ☐ no	☐ yes ☐ no	☐ yes ☐ no	☐ yes ☐ no	☐ yes ☐ no	☐ 0 days, ☐ 1-7 days, ☐ 8-30 days ☐ > 30 days
	**KNEES**	☐ yes ☐ no	__years	☐ yes ☐ no	☐ yes ☐ no	☐ yes ☐ no	☐ yes ☐ no	☐ yes ☐ no	☐ yes ☐ no	☐ 0 days, ☐ 1-7 days, ☐ 8-30 days ☐ > 30 days
	**ANKLES/FEET**	☐ yes ☐ no	__years	☐ yes ☐ no	☐ yes ☐ no	☐ yes ☐ no	☐ yes ☐ no	☐ yes ☐ no	☐ yes ☐ no	☐ 0 days, ☐ 1-7 days, ☐ 8-30 days ☐ > 30 days

		Ali ste kadarkoli imeli težave ***(kot na primer bolečina, neudobje, otopelost)*** *v predelu:*	Koliko ste bili stari, ko se je težava pojavila prvič?	Ali ste imeli v preteklih 12-ih mesecih kdaj težave v predelu:	Ali so vam težave v zadnjih 12-ih mesecih kdaj preprečile opravljanje vsakodnevnih aktivnosti (npr. hobiji, delo, pospravljanje):	Ali ste v preteklih 12-ih mesecih obiskali zdravnika zaradi težav v predelu:	Ste jemali zdravila zaradi težav v predelu:	Ali ste imeli v preteklih 7-ih dneh kdaj težave v predelu:	Ali imate danes težave v predelu:	Kolikšno je bilo najdaljše trajanje neprekinjene bolečine:
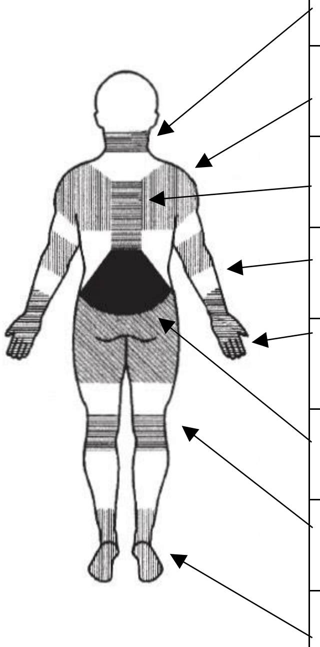	**VRAT**	☐ Da ☐ Ne	____let	☐ Da ☐ Ne	☐ Da ☐ Ne	☐ Da ☐ Ne	☐ Da ☐ Ne	☐ Da ☐ Ne	☐ Da ☐ Ne	☐ 0 dni, ☐ 1-7 dni, ☐ 8-30 dni ☐ > 30 dni
	**RAMENA**	☐ Da ☐ Ne	____let	☐ Da ☐ Ne	☐ Da ☐ Ne	☐ Da ☐ Ne	☐ Da ☐ Ne	☐ Da ☐ Ne	☐ Da ☐ Ne	☐ 0 dni, ☐ 1-7 dni, ☐ 8-30 dni ☐ > 30 dni
	**ZG. DEL HRBTA**	☐ Da ☐ Ne	____let	☐ Da ☐ Ne	☐ Da ☐ Ne	☐ Da ☐ Ne	☐ Da ☐ Ne	☐ Da ☐ Ne	☐ Da ☐ Ne	☐ 0 dni, ☐ 1-7 dni, ☐ 8-30 dni ☐ > 30 dni
	**KOMOLCA**	☐ Da ☐ Ne	____let	☐ Da ☐ Ne	☐ Da ☐ Ne	☐ Da ☐ Ne	☐ Da ☐ Ne	☐ Da ☐ Ne	☐ Da ☐ Ne	☐ 0 dni, ☐ 1-7 dni, ☐ 8-30 dni ☐ > 30 dni
	**ZAPESTJI/ROKI**	☐ Da ☐ Ne	____let	☐ Da ☐ Ne	☐ Da ☐ Ne	☐ Da ☐ Ne	☐ Da ☐ Ne	☐ Da ☐ Ne	☐ Da ☐ Ne	☐ 0 dni, ☐ 1-7 dni, ☐ 8-30 dni ☐ > 30 dni
	**SPODNJI DEL HRBTA**	☐ Da ☐ Ne	____let	☐ Da ☐ Ne	☐ Da ☐ Ne	☐ Da ☐ Ne	☐ Da ☐ Ne	☐ Da ☐ Ne	☐ Da ☐ Ne	☐ 0 dni, ☐ 1-7 dni, ☐ 8-30 dni ☐ > 30 dni
	**KOLENI**	☐ Da ☐ Ne	____let	☐ Da ☐ Ne	☐ Da ☐ Ne	☐ Da ☐ Ne	☐ Da ☐ Ne	☐ Da ☐ Ne	☐ Da ☐ Ne	☐ 0 dni, ☐ 1-7 dni, ☐ 8-30 dni ☐ > 30 dni
	**GLEŽNJA STOPALI**	☐ Da ☐ Ne	____let	☐ Da ☐ Ne	☐ Da ☐ Ne	☐ Da ☐ Ne	☐ Da ☐ Ne	☐ Da ☐ Ne	☐ Da ☐ Ne	☐ 0 dni, ☐ 1-7 dni, ☐ 8-30 dni ☐ > 30 dni
